# Identification of Sex-Related Genes from the Three-Spot Swimming Crab *Portunus sanguinolentus* and Comparative Analysis with the Crucifix Crab *Charybdis feriatus*

**DOI:** 10.3390/ani11071946

**Published:** 2021-06-29

**Authors:** Yin Zhang, Khor Waiho, Mhd Ikhwanuddin, Hongyu Ma

**Affiliations:** 1 Guangdong Provincial Key Laboratory of Marine Biotechnology, Shantou University, Shantou 515063, China; zhangyin@stu.edu.cn; 2 STU-UMT Joint Shellfish Research Laboratory, Shantou University, Shantou 515063, China; waiho@umt.edu.my (K.W.); ikhwanuddin@umt.edu.my (M.I.); 3 Higher Institution Centre of Excellence (HICoE), Institute of Tropical Aquaculture and Fisheries, Universiti Malaysia Terengganu, Kuala Terengganu 21030, Malaysia

**Keywords:** *Portunus sanguinolentus*, comparative transcriptome, differentially expressed genes, functional annotation

## Abstract

**Simple Summary:**

Crabs within the family Portunidae are important marine species in both aquaculture and fishery sectors. The current aquaculture status of most portunids still relies on wild-caught fisheries due to the lack of essential knowledge regarding their reproductive biology and underlying governing mechanism. In the present study, we compared the differentially expressed genes (DEGs) between the different sexes of *Portunus sanguinolentus* based on their gonadal transcriptome profiles and subsequently contrasted them with the gonadal DEGs of *Charybdis feriatus*, the other member of the family Portunidae. In total, 40,964 DEGs between the ovaries and testes of *P. sanguinolentus* were uncovered, with 27,578 up-regulated and 13,386 down-regulated in females. After comparison, *C. feriatus* has approximately 63.5% of genes in common with *P. sanguinolentus*, with 62.6% showing similar expression patterns. Interestingly, the *DMRT* gene was specifically expressed in male *P. sanguinolentus*, while its homologous gene—*doublesex* (*DSX*)—was specifically expressed in male *C. feriatus*. The DEGs obtained from the gonadal transcriptome of *P. sanguinolentus* are a beneficial resource for future genetic and genomic research in *P. sanguinolentus* and its close species. The transcriptomic comparison analysis might provide references for better understanding the sex determination and differentiation mechanisms among portunids.

**Abstract:**

Crabs within the family Portunidae are important marine species in both aquaculture and fishery sectors. The current aquaculture status of most portunids, however, still relies on wild-caught fisheries due to the lack of essential knowledge regarding their reproductive biology and underlying governing mechanism. With the advancement of sequencing technology, transcriptome sequencing has been progressively used to understand various physiological processes, especially on non-model organisms. In the present study, we compared the differentially expressed genes (DEGs) between sexes of *Portunus sanguinolentus* based on their gonadal transcriptome profiles and subsequently contrasted them with the gonadal DEGs of *Charybdis feriatus*, the other member of Family Portunidae. In total, 40,964 DEGs between ovaries and testes were uncovered, with 27,578 up- and 13,386 down-regulated in females. Among those, some sex-related DEGs were identified, including a *dmrt-like* (*DMRT*) gene which was specifically expressed in males. *C. feriatus* has approximately 63.5% of genes common with *P. sanguinolentus*, with 62.6% showing similar expression patterns. Interestingly, the *DMRT* gene was specifically expressed in male *P. sanguinolentus* while its homologous gene—*doublesex* (*DSX*)—was specifically expressed in male *C. feriatus*. The DEGs obtained from the gonadal transcriptome of *P. sanguinolentus* are a beneficial resource for future genetic and genomic research in *P. sanguinolentus* and its close species. The transcriptomic comparison analysis might provide references for better understanding the sex determination and differentiation mechanisms among portunids.

## 1. Introduction

Portunidae are important marine crab species with a diversity and speciation of around 300 members [1], among which many are considered important marine aquaculture and captured-fishery species [2]. The culture of crabs has been gaining attention since the last decade because of the high demand for live crabs and crab products in the global market. In particular, *Portunus sanguinolentus* and *Charybdis feriatus* are potential marine aquaculture species in China due to their high meat quality and fast growth rate [3]. However, the current crab culture is still largely dependent on wild capture fishery [4]. The unregulated fisheries practice, combined with increasing overfishing and environmental deterioration, resulted in the quick depletion of many crab resources [5]. *P. sanguinolentus* is generally named the three-spot swimming crab for its three distinct red to chestnut spots on the back of its carapace. It can be found in Indo-Pacific waters from the east coast of South Africa to Hawaii [6]. So far, genetic studies on *P. sanguinolentus* are limited. Thus, the availability of *P. sanguinolentus* gonadal transcriptomic profiles will benefit greatly to the revealing of the regulatory roles of ovary and testis in portunids and serve as a useful comparison for other marine crabs.

*Charybdis feriatus*, naturally distributed in the coastal areas of Guangdong, Zhejiang, Guangxi, Hainan, and Fujian provinces of China and the waters of Indo-Pacific regions, is a large, highly prized marine portunid crab species. Juvenile crabs inhabit sandy shores while adults preferentially move to the muddy offshore areas [7]. They are easy to distinguish according to their prominent red and white carapace and an obvious cross on the median surface of their carapace [8]. Despite belonging to different genera, *P. sanguinolentus* and *C. feriatus* share not just morphological similarities, but also life cycles and regeneration ability. However, the information of a genome, transcriptome, and associated molecular markers of these two species are rare at the moment.

RNA-seq is a practical tool for uncovering differential gene expression under certain conditions of non-model organisms. It has been successfully performed in many aquaculture invertebrates for various purposes, including identification of differentially expressed genes (DEGs) in different tissues or under specific conditions in crabs like *Scylla paramamosain* [9,10], *Scylla olivacea* [4], *Portunus trituberculatus* [11,12], *Eriocheir sinensis* [13,14] and *Sinopotamon henanensis* [15]. RNA-seq analysis is also used in resolving comparative genomic-level issues, especially for non-model organisms [16]. In principle, comparative RNA-seq studies between closely related species could offer excess genomic resources and simultaneously supply information about the processes of differentiation between species [17].

The present study aimed at screening the DEGs between sexes of *P. sanguinolentus* based on its gonadal transcriptome data. In addition to contributing to the genetic resources available for portunid species, we compared the gonadal DEGs of *P. sanguinolentus* with that of *C. feriatus* obtained from previous research [18] in an attempt to understand the similarities and differences in sex-biased genes of both portunid species. These data would be helpful for future studies on the gonad molecular regulatory mechanism and sex differentiation mechanism of marine crabs.

## 2. Materials and Methods

### 2.1. Transcriptome Analysis and Validation

The present experiment was approved by the Institutional Animal Care and Use Ethics Committee of Shantou University. The detailed transcriptome analysis method was described in [19]. The ovaries in stage III-IV and testes in stage II-III without vas deferens were extracted from four females and four males *P. sanguinolentus*, respectively. Females *P. sanguinolentus* were named as PS-F and males as PS-M. In brief, total RNA was extracted from the gonads of PS-F and PS-M, converted into cDNA libraries, and sequenced on Illumina HiSeq 3000 platform. Clean reads were subjected to *de novo* assembly and annotated to public databases.

### 2.2. Analysis of Differentially Expressed Genes of P. sanguinolentus

StringTie was used to calculate FPKMs (Fragment per kilobase of exon model per million mapped reads) [20] of transcripts of the testes (PS-M) and ovaries (PS-F) samples of *P. sanguinolentus*. FPKMs were calculated based on the length of the fragments and reads count mapped to this fragment. Prior to differential gene expression analysis, for each sequenced library, the read counts were adjusted by edgeR program package through one scaling normalized factor. DEGs were detected by the EBSeq package with raw counts as inputs. Unigene with false discovery rate (FDR) ≤ 0.001 and the absolute value of log2 Ratio ≥ 1 was considered significantly different expressed gene. GOseq with the Wallenius non-central hyper-geometric distribution model was used for Gene Ontology (GO) enrichment analysis (*p*-value ≤ 0.05), and Kyoto Encyclopedia of Genes and Genomes (KEGG) pathway enrichment analysis using KOBAS with the hyper-geometric distribution model.

### 2.3. RNA-Seq Results Verification

Nineteen genes, i.e., one male-specific expressed gene *DMRT*, three up-regulated genes in male (*male reproductive-related LIM protein* (*MRLIM*), *male sterility domain-containing protein* (*MSD*), myosin heavy chain (*MHC)*), and 15 up-regulated genes in female (*androgen-induced protein 1* (*AIP*), *cyclin B* (*CYCB*), *estradiol receptor-like protein* (*ER*), *extra sex combs* (*ESC*), *fem-1-like protein* (*FEM1*), *heat shock protein 70* (*HSP70*), *HSP90*, *juvenile hormone esterase* (*JHE*), *ovarian fibroin-like substance-2* (*OFLS*), *progesterone-like protein* (*PG*), *vasa-like* (*VASA*), *vitellogenin* (*VTG*), *vitellogenin receptor* (*VTGR*), *ovary development-related protein* (*ODR*), *sox14 protein* (*SOX14)*), were selected for RNA-seq verification using quantitative real-time PCR (qPCR). cDNA was reverse transcripted from 1 μg total RNA using Reverse Transcription System (Transgen Biotech Co. Ltd., Beijing, China). Primer 6.0 Software was used to design the primers (Supplementary Table S1) for qPCR which is performed in a Mini Option real-time detector (Roche Light Cycle@480). The reaction solution for qPCR included 2.0 μL cDNA solution (20 ng), 0.6 μL PCR forward primer (10 μM), 0.6 μL PCR reverse primer (10 μM), 10 μL Talent qPCR Premix (2×) (TIANGEN Biotech Co., Ltd., Beijing, China), and 6.8 μL RNase-free water. The reaction conditions were performed following the recommendation of the instruction. All amplicons were initially separated by agarose gel electrophoresis to ensure their sizes. The expression level of each gene was normalized towards the reference gene (*18s rRNA*). Gene expression levels were calculated using the optimized comparative Ct (2^−ΔΔCt^) value method.

### 2.4. Comparison of the DEGs between P. sanguinolentus and C. feriatus

Using the previously published data of *C. feriatus* (CF) [18], the whole gonadal transcriptome profiles between the two crab species were compared using BLAST. The assembled sequences from the two species were considered as the same transcriptome and given a new ID considering as “new unigene” when the identity reached 98%. At least one transcript was mapped to one “new unigene” in both libraries. Raw fragments detected in each sample of the two profiles and FPKM values were accordingly considered as the FPKM value of the “new unigene”. If the new unigene is composed of more than one transcript, the FPKM values of more than one transcript are added together as the FPKM of the “new unigene”. Subsequently, the expression values (FPKM) of the “new unigenes” among females (PS-F vs. CF-F) and males (PS-M vs. CF-M) of the two portunid species were compared, respectively. The threshold of the DEGs was set as FDR < 0.001 and the absolute value of log2 Ratio > 1. The GO and KEGG annotations of the “new unigenes” were performed following Section 2.2.

## 3. Results

### 3.1. DEGs of P. sanguinolentus

**Figure 1:**
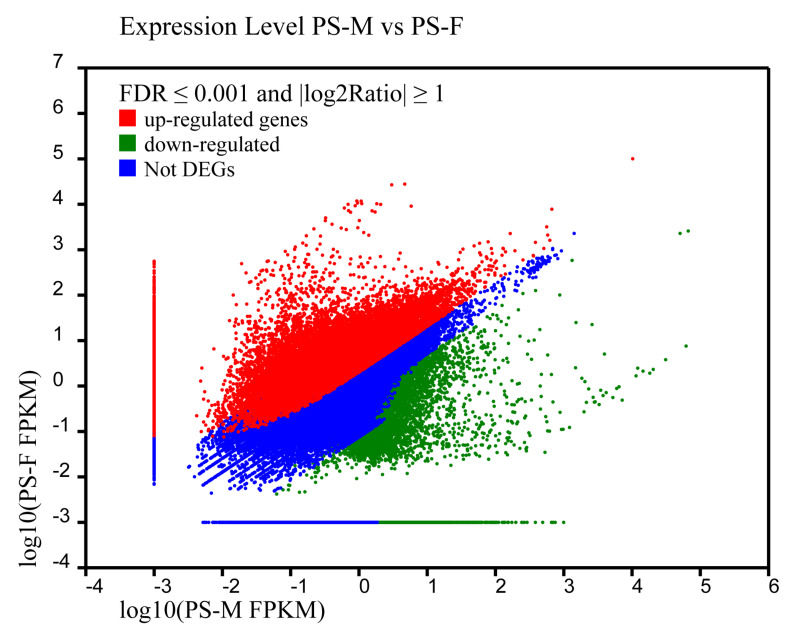
The genes were classified into three classes. Blue genes are not differentially expressed genes. Green genes are down-regulated that gene expression of left sample is larger than right sample. Red genes are up-regulated that gene expression of right sample is larger than left sample. The horizontal coordinate is the expression level of PS-M and the vertical coordinates is the expression level of PS-F. PS-M represents the male *Portunus sanguinolentus* and PS-F represents female *Portunus sanguinolentus*. *n* = 4 for each sex.

To identify the DEGs between males and females of *P. sanguinolentus*, the mapped reads were normalized to calculate the unigenes expressions between the PS-M and PS-F using FPKM value. The details of unigene expressions can be found in Supplementary Table S2. Among all unigenes, 40,964 DEGs were obtained with 27,578 up- and 13,386 down-regulated in female *P. sanguinolentus* (Figure 1, Supplementary Table S3). Some genes directly or indirectly related to sex determination and differentiation, like *AIP*, *CYCB*, *DmX-like* (*DMX*), *ER*, *FEM1*, *GPR*, *HSP90*, *PG*, *wnt4 precursor* (*WNT4P*), *VASA*, *VTG*, *VTGR*, *crustacean hyperglycemic hormone (CHH)* and *ovary development-related protein* (*ODR*) were up-regulated in females, while male-reproductive-related genes such as *Dmc1-like* (*DMC1*), *MRLIM*, *MSD*, *sex peptide receptor* (*SPR*), *insulin receptor* (*IR*) and *spermatogenesis-associated protein 2* (*SPATA2*) were up-regulated in males. Besides, *SRY-like* (*SRY*) and *dmrt-like* (*DMRT*) gene were specifically expressed in males.

**Figure 2:**
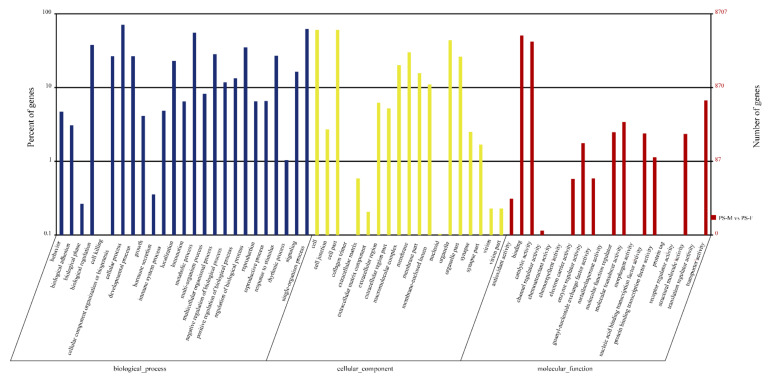
Gene ontology (GO) assignment of differentially expressed genes (DEGs) of *Portunus sanguinolentus*. GO functions is showed in *X*-axis. The *Y*-axis shows the percent and number of genes which have the GO function.

These DEGs were further analyzed using KEGG pathway enrichment to determine their metabolic pathways. The significant enrichment KEGG pathway (*p* value < 0.0001) were Ribosome biogenesis in eukaryotes, Aminoacyl-tRNA biosynthesis, Parkinson’s disease, Proteasome as well as Glyoxylate and dicarboxylate metabolism (Figure 2).

**Figure 3:**
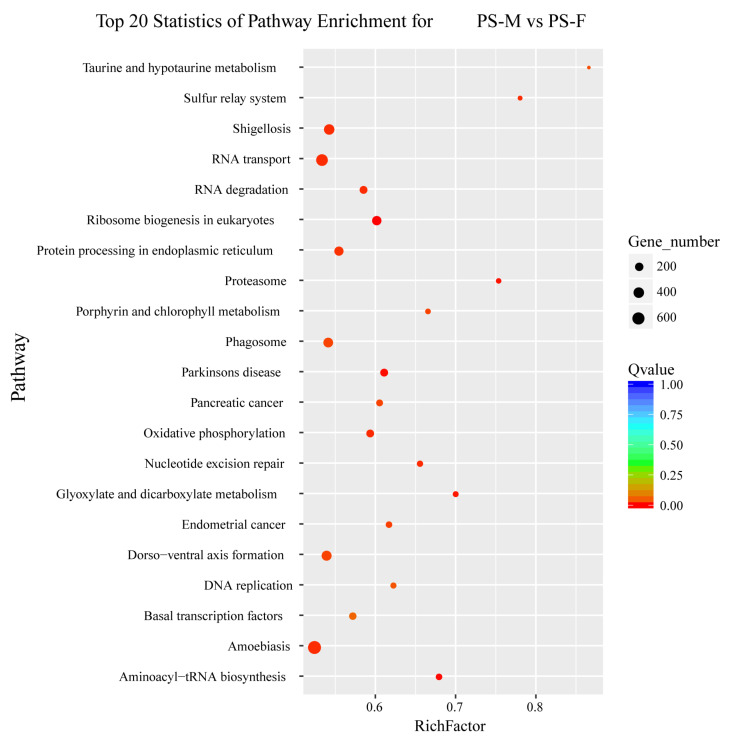
Top 20 statistics of pathway enrichment for PS-M vs. PS-F. PS-M represents the male *Portunus sanguinolentus* and PS-F represents female *Portunus sanguinolentus*.

**Figure 4:**
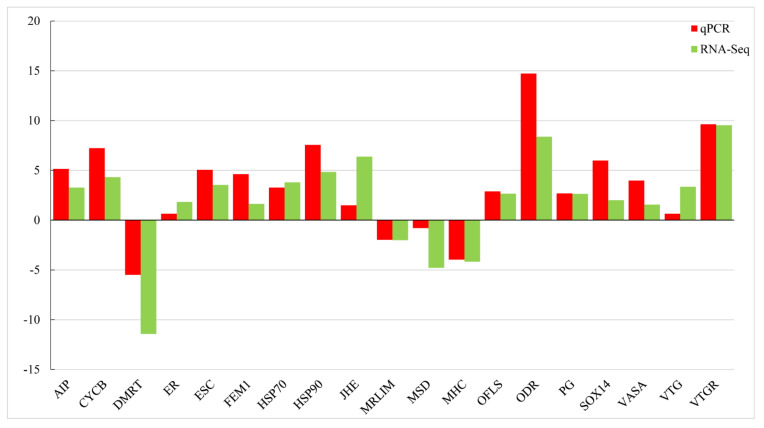
Candidate unigene expression levels revealed by qRT-PCR (red bar) and RNA-seq (green bar). *n* = 4 for each sex, the results of qRT-PCR were performed by relative expression using *18S rRNA* as the reference gene and measured by the method of optimized comparative Ct (2^−ΔΔCt^) value, the qRT-PCR results were performed by log_2_ (female/male) and the RNA-seq results by log_2_ (female FKPM/male FKPM). They are showing the same expression trend.

The DEGs obtained from the comparison of PS-F and PS-M were subjected to GO annotation to see their potential functions. In the biological process, cellular process (6143 DEGs), single-organism process (5418 DEGs), metabolic process (4803 DEGs), biological regulation (3280 DEGs), and multicellular organismal process (3034 DEGs) were the most significant enrich GO function items in the comparison while cell (5268 DEGs), cell part (5261 DEGs), organelle (3819 DEGs), membrane (2617 DEGs), organelle part (2287 DEGs) were the most in a cellular component. Followed binding which was the most significant abundant GO function items with 4426 DEGs, catalytic activity (3630 DEGs), transport activity (580 DEGs), molecular transducer activity (298 DEGs), and molecular function regulation (216 DEGs) were more significant enrich GO items than the others in the classify of molecular function (Figure 3). The similar expression patterns of the 19 genes between qPCR and RNA-Seq verified the results of the mRNA-seq (Figure 4).

### 3.2. Comparison of Unigenes between P. sanguinolentus and C. feriatus

**Figure 5:**
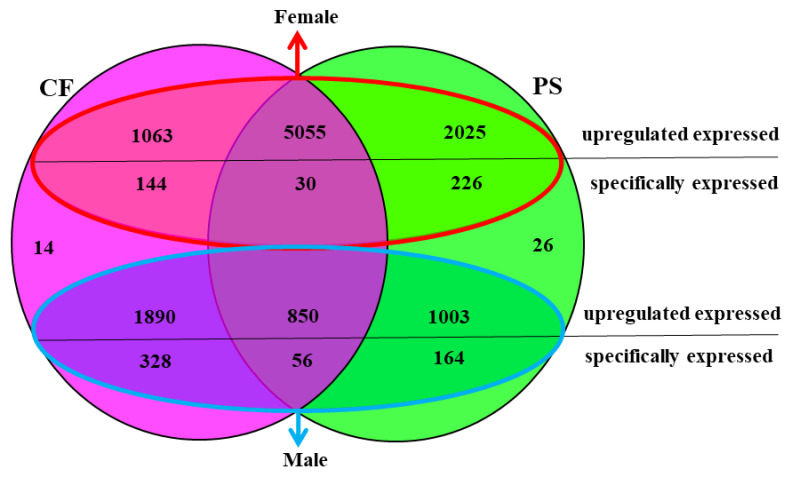
A Venn diagram showing the number of unigenes comparing PS vs. CF. The circle with the red border refers to the females and the blue refers to the males. PS represents *Portunus sanguinolentus* and CF represents *Charybdis feriatus*. The numbers of the unigenes represent all the new unigenes detected in the present study.

**Figure 6:**
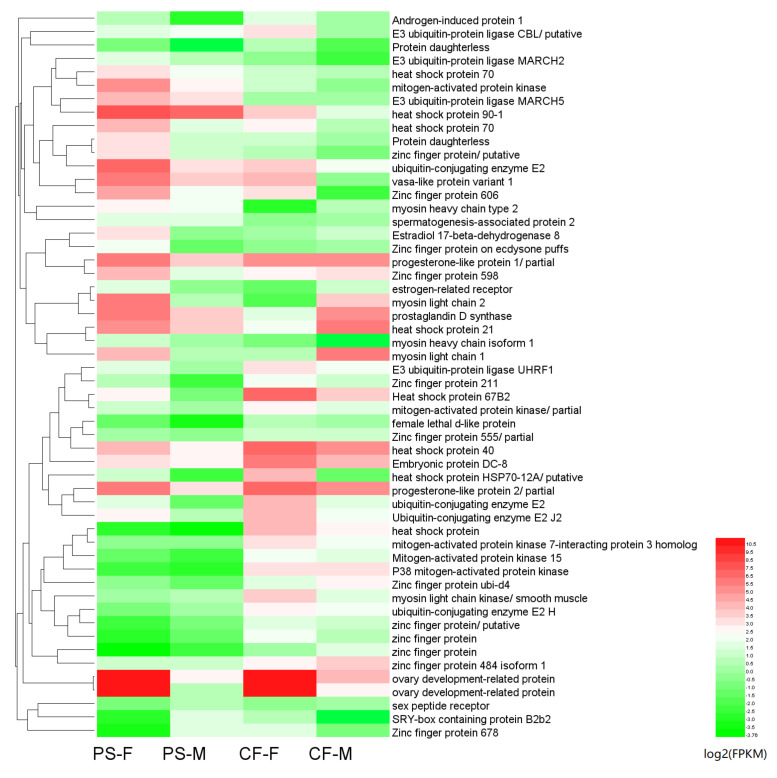
Heat map of DEGs related with sex differentiation and determination co-expressed in males and females *Portunus sanguinolentus* and *Charybdis feriatus*. PS-M represents the male *Portunus sanguinolentus* and PS-F represents female *Portunus sanguinolentus*. CF-M represents the male *Charybdis feriatus* and CF-F represents female *Charybdis feriatus*.

**Figure 7:**
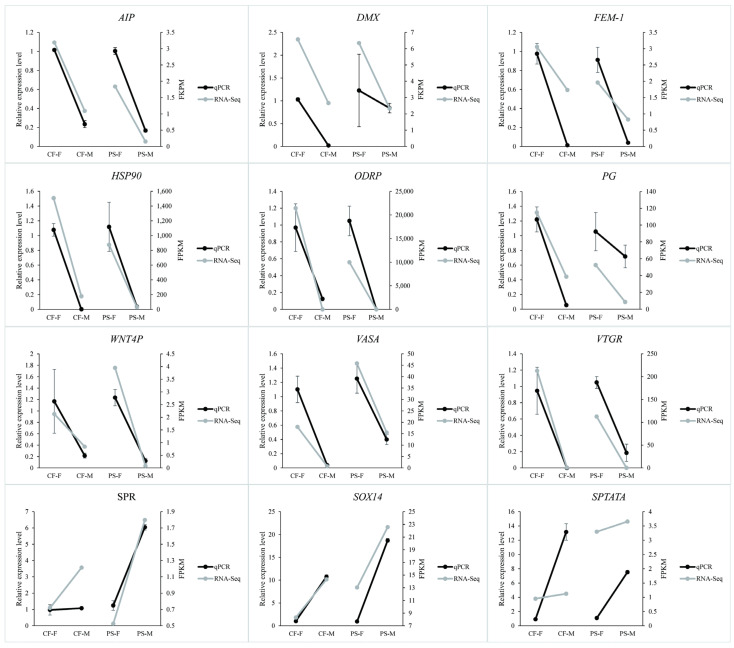
The verification of the genes which have the same expression patterns between sexes of *Portunus sanguinolentus* and *Charybdis feriatus*. PS-M represents the male *Portunus sanguinolentus* and PS-F represents female *Portunus sanguinolentus*. CF-M represents the male *Charybdis feriatus* and CF-F represents female *Charybdis feriatus*.

The expression levels (FPKM values) of the unigenes in both libraries were mostly (~99%) between 0–100 (Table 1). 9,436 “new unigenes” with more than 98% similar identities on both libraries (Table 2, Supplementary Table S4) were identified. Among the 9,436 unigenes, approximately 63.5% of genes were shared between the libraries of *P. sanguinolentus* and *C. feriatus*, with 62.6% showed similar expression patterns. More DEGs were shared among females (*n* = 5085) of the two species compared to males (*n* = 906). The number of upregulated (*n* = 7080) and specifically expressed (*n* = 256) DEGs were higher in *P. sanguinolentus* females when compared to *C. feriatus* females. In contrast, the males of *C. feriatus* had higher upregulated and specifically expressed DEGs compared to those of *P. sanguinolentus* (Figure 5). Some genes related to sex determination and differentiation in *P. sanguinolentus* and *C. feriatus* were screened out from the shared up- and down-regulated DEGs between the two portunid species (Figure 6), such as *AIP*, *ODR*, *CYCB*, *DMX*, *GPR*, *PG*, *WNT4P*, *VASA*, and *VTGR* were up-regulated in females, and some were up-regulated in males including *SPR* and *SPATA2*. We also verified those genes with the same expression tendency in both *P. sanguinolentus* and *C. feriatus*, revealing the same expression patterns with RNA-seq (Figure 7). Additionally, *DMRT* was specifically expressed in PS-M while no *DMRT* gene was found to be differentially expressed in *C. feriatus*, but its homologous gene *DSX* was specifically expressed in CF-M.

**Figure 8:**
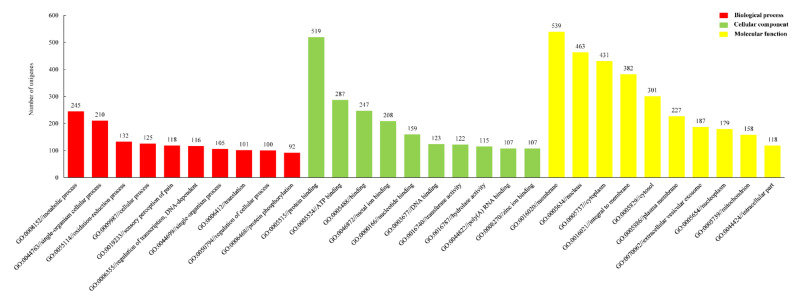
Gene ontology (GO) enrichment of new unigenes with >98% identity between two libraries of PS and CF. PS represents *Portunus sanguinolentus* and CF represents *Charybdis feriatus*.

The “new unigenes” were annotated against the GO database, respectively (Figure 8). In the biological process, metabolic process (GO: 0008152, 245 unigenes), single-organism cellular process (GO: 0044763, 210 unigenes), oxidation-reduction process (GO: 0055114, 132 unigenes), sensory perception of pain (GO: 0019233, 118 unigenes) and regulation of transcription, DNA-dependent (GO: 0006355, 116 unigenes) were the most enrichment items while protein binding (GO: 0005515, 519 unigenes), ATP binding (GO: 0005524, 287 unigenes), binding (GO: 0005488, 247 unigenes), metal ion binding (GO: 0046872, 208 unigenes), nucleotide-binding (GO: 0000166, 159 unigenes) were the most in the classify of molecular function. In cellular component, membrane (GO: 0016020, 539 unigenes), nucleus (GO: 0005634, 463 unigenes), cytoplasm (GO: 0005737, 431 unigenes), integral to the membrane (GO: 0016021, 382 unigenes), cytosol (GO: 0005829, 301 unigenes) enriched most unigenes.

## 4. Discussion

### 4.1. DEG Profiles of P. sanguinolentus

High-throughput transcriptome sequencing allows analysis of DEGs under different physiological conditions and treatments [21,22]. Our previous study identified a total of 119,718 unigenes with an average length of 904 nt in *P. sanguinolentus* [19]. As a continuation, in the present study, an analysis of DEGs in male and female *P. sanguinolentus* was performed, aiming to explore potentially valuable genes to better comprehend the basics of sex determination and differentiation biological mechanisms in *P. sanguinolentus* and possibly serve as references for other crustacean species. A comprehensive grasp of the molecular mechanisms of sex preference and the regulatory pathways that occur in the gonads may lead to future manipulation of desired traits and will impart data resources for future gene expression, functional and reproductive investigation for *P. sanguinolentus*.

Comparing between sexes, the expression of upregulated unigenes in *P. sanguinolentus* ovary was almost twice that of the testis. This expression pattern of more transcripts upregulated in females was also found in the comparative gonadal transcriptome of other invertebrates, such as in the gonadal transcriptome of the *S. paramamosain* [23,24] and *C. feriatus* [18], as well as crustacean *Caligus rogercresseyi* [25]. This pattern of expression might be due to the complex role of the ovary in the female reproduction process. After DEG annotation, most DEGs were involved in ribosome biogenesis in eukaryotes (*p* value: 2.81 × 10^−5^). Ribosomes are the workplace for protein biosynthesis and are directly associated with translation, localization, and cell growth, cycle, and proliferation [26]. The high enrichment of DEGs in ribosome biogenesis could be due to the different reproduction-related processes occurring within the testis and ovary of *P. sanguinolentus*, including gametogenesis and hormonal regulation. Additionally, growth-related GO items such as cellular process, cell, binding in the classify of biological process, cellular component, and molecular function were found to be enriched based on the DEGs of *P. sanguinolentus*. A similar pattern was detected in the gonadal DEGs of *S. paramamosain* [10] and *E. sinensis* [27], highlighting the potential involvement of a high number of sex-biased genes in the gonadal growth and development of portunid species.

### 4.2. Genes Differentially Expressed in Females and Males of P. sanguinolentus

The FPKMs of the unigenes obtained from gonads of *P. sanguinolentus* were mainly in the range of 0–100, which is accordant with *C. feriatus.* However, several unigenes exhibited FPKM values beyond ten thousand, among which was the *ODR* gene, specifically expressed in females of *P. sanguinolentus* and *C. feriatus*, suggesting that the ODR were vital for female reproductive development. Additionally, as expected, *PG* and *VTGR* were upregulated in both females of *P. sanguinolentus* and *C. feriatus.* The former is involved in oogenesis and ovarian development and maturation of animals including crustaceans [28,29] while the latter existed in the oocyte membranes [30]. PGs participate in regulating the oocyte maturation of animals including crustaceans [28,31,32]. And it has been reported to stimulate ovarian maturation in *Parapanaeopsis hardwickii* [33]. Furthermore, PG can also promote the synthesis of *VTG* mRNA, as reported in freshwater crayfish *Cherax albidus* [34]. The female up-regulated *VTG* gene is crucial to the development of oocyte and embryo [35]. VTGs were up-regulated in the mature female gonad of *P. trituberculatus* [36], suggesting that the up-regulation of *VTG* might be a preparation for oocyte development. Additionally, estrogen hormones may also increase the synthesis of VTG in oviparous vertebrates by the increase in *VTG* gene transcription, and the estrogen hormone is regulated by *HSP90* [37,38]. Paolucci et al. [39] showed that the presence and up-regulation of *PG* and *ER* resulted in the accumulation of VTG in crayfish. A previous study also showed that *HSP90* was involved in the regulation of VTG synthesis [38] and was upregulated in the copulated ovary of *D. melanogaster* [40]. In the present study, *PG*, *VTG*, *HSP90*, and *ER* genes were differentially expressed in our transcriptomic sequencing data, with a higher level of expression in ovaries than in testes. This indicates the involvement of *PG*, *VTG*, *HSP90*, and *ER* genes in the oocyte development of crabs and highlights the conserved oogenesis maturation pathway in animals.

Specifically expressed in the gonads, the *VASA* gene is actively expressed during early gametogenesis [41,42]. VASA exists in both invertebrates and vertebrates [41] and plays a vital role in germ cell development, proliferation, maintenance, and gametogenesis [41], including the gonadal development and gametogenesis of crustaceans [42,43,44]. Specifically, the *VASA* gene is expressed in germ cells encoding for RNA-dependent helicase during the whole developmental stages of pacific white shrimp *Litopenaeus vannamei* [43]. The present study found that the *VASA* gene was up-regulated in ovaries, which is in accordance with that in *S. paramamosain* [23] and *C. feriatus* [18], verifying its reproduction-related function. And it was found that Vasa mainly function in auxiliary cells of oyster ovaries [45].

The upregulation of *CYCB* in the females of *P. sanguinolentus* and *C. feriatus* was expected as cyclin B was commonly known to be related to the meiotic maturation of oocytes [46] and active during crab’s ovarian maturation [46]. In line with the results of this study, a high expression level of *CYCB* in the ovary was also observed in *S. paramamosain* [47], *E. sinensis* [48], *Macrobrachium rosembergii* [49], and *P. monodon* [46], suggesting that cyclin B is crucial for the ovarian development and maturation of crustaceans.

Some female up-regulated genes in *P. sanguinolentus* that were potentially involved in the regulation of gametogenesis, reproduction, gonadal differentiation, and development, such as *FEM-1* [11], *CHH* [50], and *GPR* [23] were identified in this study. It is reported that FEM-1 might be involved in the early sex determination and late gonad development of crabs [51]. CHH may be involved in the inhibition of molting and ovarian development of crabs [52,53]. GPR is involved in the reproductive system’s maturation [23]. *WNT4P* was also found to be upregulated in females. WNT4, a member of the WNT family, plays a vital role in female reproductive development in mammals, controlling steroidogenesis in the gonad and supporting oocyte development [54,55]. Lack of *WNT4* in mice led to a striking reduction in the number of developing oocytes and resulted in gonadal masculinization of the female [56]. Besides, *DMX*, which contains a DNA-binding motif called DM domain, was up-regulated in both males of *P. sanguinolentus* and *C. feriatus*, which might be potentially related to the sex determination mechanisms of the two species. The up-regulated gene *AIP* was a useful probe for studies on specific gene expression and prostatic secretory mechanisms regulated by androgen [57].

Additionally, some sex determination and differentiation-related genes were found to be upregulated in males, including *DMC1*, *MRLIM1*, *MSD*, *SPR*, and *SPATA2*. The up-regulated expression of *DMC1* in male *P. sanguinolentus* was in accordance with that in the male giant tiger shrimp *Penaeus monodon* [58]. *DMC1* was specifically expressed in embryonic ovaries and testicular germ cells during meiosis of Chinese mitten crab [59]. A similar up-regulation pattern of *MRLIM1* in males of both *P. sanguinolentus* and *C. feriatus* was also observed in the oriental river prawn *Macrobrachium nipponense* [60]. The MRLIM1 was reported to be involved in the endocrinal function of the eyestalk [61]. *MSD* might be related to male sterility. *SPR*, upregulated in both males of *P. sanguinolentus* and *C. feriatus*, was also expressed in embryonic and larval stages of *Drosophila* as well as in the adult male nervous system, while the expression of *SP* was confined to the male reproductive system [62]. Maurizio et al. [63] reported that *SPATA2* was expressed in testis of rats and originated from Sertoli cells in the period of infantile-juvenile and increases gradually during growing up. Some genes like *IR*, *SRY*, and *DMRT* were specifically expressed in males. IR was found to be the receptor of insulin-like androgenic gland hormone (IAG) [64]. IAG is regarded as the key regulator in crustacean male sexual differentiation [65]. *SRY* was discovered in the human Y chromosome for the first time as a sex-determining factor and contains a conserved high-mobility group (HMG) box [66], *SOX14* was similar to the HMG box with *SRY* on sequences [67].

In short, some genes showed similar upregulation patterns in both females and males of *P. sanguinolentus* and *C. feriatus*. This might signify the close relationship between the two species and indicate that the genes related to sex determination and differentiation mechanisms were relatively conservative between portunids. The GO annotation of the “new unigenes” indicated that metabolic process, protein binding, and membrane were respective enriched most amount of unigenes, which was accordance with the GO annotation of total unigenes in *P. sanguinolentus* [19], *C. feriatus* [18], *E. sinensis* [26], *S. paramamosain* [23] and *P. trituberculatus* [11].

### 4.3. Differences between the DEGs of P. sanguinolentus and C. feriatus

When comparing the DEGs of *P. sanguinolentus* and *C. feriatus*, the *DMRT* gene was specifically expressed in male *P. sanguinolentus*, whereas its homologous gene *DSX* gene was specifically expressed in male *C. feriatus* instead of the *DMRT* gene. DMRT and DSX belong to the same DM DNA-binding domain superfamily [68]. *DMRT*, which has a function in sex differentiation and reproductive development, was also found to be differentially expressed in other crabs [11,69] but not in *E. sinensis* [26] and *S. paramamosain* [10]. Whereas, *DSX* gene functions in the sexual differentiation of crustaceans and is indispensable for male trait development [70]. The species-specific expression pattern here might be related to the different sex determination regulation mechanisms and the DM domain gene evolutions in different species, which warrants further research. *DMRT1* is one of the well-conserved genes associated with sex differentiation from invertebrate to vertebrate, despite the great diversity of animal sex-determination mechanisms [71]. The difference in the expression of *DMRT* and *DSX* between the two species in our study was speculated on the account of the divergence of sex determination mechanisms between *P. sanguinolentus* and *C. feriatus*, although future validation is urgently needed to support this hypothesis.

## 5. Conclusions

The present study provides a comprehensive excavation of DEGs of *P. sanguinolentus* through the comparison of its gonadal transcriptome profiles. As a whole, functional analyses of the present dataset identified many gonadal DEGs (such as female up-regulated genes including *AIP*, *ODR*, *CYCB*, *DMX*, *GPR*, *PG*, *WNT4P*, *SOX21b*, *VASA*, *CHH*, and *VTGR*, male up-regulated genes including *DMC1*, *MRLIM*, *MSD*, *SPR*, *SPATA2*, *IR*, and *SRY*) that are potentially involved in reproduction, specifically sex determination and differentiation. In addition, we found that the *DMRT* gene was specifically expressed in male *P. sanguinolentus* and the *DSX* gene specifically in male *C. feriatus*. We proposed to focus on sex determination mechanisms of *P. sanguinolentus* and *C. feriatus* in future work to verify the difference revealed in the present study. Our results illustrate the application of high throughput transcriptome sequencing as a basis for mining tissue specific functional genes in a non-model species. The mine of sex-related genes from gonads of *P. sanguinolentus* in the present study will be helpful for the study of the mechanism of sex determination and differentiation as a reference for crabs. These transcriptome data will contribute to future gene functional and genomic analysis of portunid species.

## Figures and Tables

**Table 1:** The distribution of genes according to the expression level FPKM.

FPKM	Gene Number of PS-F	Gene Number of PS-M	Gene Number of CF-F	Gene Number of CF-M
0–100	119,142	119,469	85,851	86,074
101–1000	521	216	507	309
1001–10,000	47	23	63	45
>10,000	8	10	12	5

PS-M represents the male *Portunus sanguinolentus* and PS-F represents female *Portunus sanguinolentus*. CF-M represents the male *Charybdis feriatus* and CF-F represents female *Charybdis feriatus*.

**Table 2:** The summary of new unigenes with >98% identity between two libraries of *Portunus sanguinolentus* and *Charybdis feriatus*.

	PS-M	PS-F	CF-M	CF-F
Up-regulated	1853	7080	2745	6118
Specifically expressed	220	256	384	174
No expression	26		14	
Total	9435			

PS-M represents the male *Portunus sanguinolentus* and PS-F represents female *Portunus sanguinolentus*. CF-M represents the male *Charybdis feriatus* and CF-F represents female *Charybdis feriatus*.

## Data Availability

The transcriptome data of *Charybdis feriatus* can be found in the NCBI Short Read Archive (SRA) database under accession SRR6214447 (https://www.ncbi.nlm.nih.gov/sra/?term=SRR6214447), and the Transcriptome Shotgun Assembly (TSA) project has been deposited at DDBJ/EMBL/GenBank under the accession GGFD00000000 (https://www.ncbi.nlm.nih.gov/nuccore/GGFD00000000). *Portunus sanguinolentus* transcriptome data was under accession SRR6216182 in SRA (https://www.ncbi.nlm.nih.gov/sra/?term=SRR6216182), and assembly sequence file was in the TSA project at DDBJ/EMBL/GenBank under the accession GFZC00000000 (https://www.ncbi.nlm.nih.gov/nuccore/GFZC00000000). The details of differentially expressed genes in *P. sanguinolentus* can be found in Supplementary Materials (https://doi.org/10.5281/zenodo.4698549).

## Supplementary Materials

The following are available online at https://www.mdpi.com/article/10.3390/ani11071946/s1, Supplementary Table S1 Primers used for qPCR. Supplementary Table S2 The expression details of all unigenes of *Portunus sanguinolentus*. Supplementary Table S3 The expression and annotations of differentially expressed genes in male and female *Portunus sanguinolentus*. Supplementary Table S4 The expressions and annotations of “new unigenes” of *Portunus sanguinolentus* and *Charybdis feriatus*.
